# Postmortem histological freeze–thaw artifacts: a case report of a frozen infant and literature review

**DOI:** 10.1007/s12024-023-00752-w

**Published:** 2023-11-20

**Authors:** Elena Giovannini, Maria Paola Bonasoni, Marcellino Bardaro, Giuseppe Russello, Edoardo Carretto, Alessandro Zerbini, Giancarlo Gargano, Susi Pelotti

**Affiliations:** 1 Present Address: Unit of Legal Medicine, Department of Medical and Surgical Sciences, University of Bologna, Via Irnerio 49, 40126 Bologna, Italy; 2 Pathology Unit, Azienda USL-IRCCS Di Reggio Emilia, Via Amendola 2, 42122 Reggio Emilia, Italy; 3 Unit of Microbiology, Azienda USL-IRCCS Di Reggio Emilia, Via Amendola 2, 42122 Reggio Emilia, Italy; 4 Unit of Clinical Immunology, Allergy and Advanced Biotechnologies, Azienda USL-IRCCS Di Reggio Emilia, Via Amendola 2, 42122 Reggio Emilia, Italy; 5 Neonatal Intensive Care, Azienda USL-IRCCS Di Reggio Emilia, Via Amendola 2, 42122 Reggio Emilia, Italy

**Keywords:** Freezing, Thawing, Histological artifacts, Ice crystals, Extracellular space expansion

## Abstract

Freezing and thawing have the potential to alter the gross and histologic appearance of tissues, causing damage to individual cells and disrupting the overall architecture. In forensic investigations, freezing and thawing can play a crucial role in cases of unknown cause of death. Perpetrators may use freezing preservation to conceal the body or obscure the time of death. Freezing can also occur naturally when a body is exposed to the elements, sometimes even leading to death itself. We present a case report involving an autopsy performed on an infant, who died of natural causes, after undergoing freezing and thawing. The objective of this study was to identify and discuss the histological artifacts observed in different tissues as a result of the freeze–thaw process. Histologically, the infant’s tissues exhibited the most common features described in the literature. Ice crystal artifacts, characterized by expansion of the extracellular space and tissue clefts, were found in the heart, brain, liver, lungs, and kidneys. On the contrary, adipose tissue was not affected, likely due to the scarcity of water. Freeze–thaw artifacts should be taken into account whether a body is known to have been frozen or to add further data if found already defrosted.

## Introduction

Freezing and thawing have the potential to alter the gross and histological appearance of tissues, damaging individual cells and disrupting the overall architecture. However, the exact mechanism and influencing factors of this damage are not yet fully understood [1].

It is believed that the formation and rupture of cell wall pores result from fluid shifts that take place during the freezing and thawing process. As the extracellular water freezes, fluid moves out from the cells and pours into the extracellular space. This movement of liquid leads to increased intracellular solute concentration, causing cell shrinkage and the disruption of cellular junctions. When the tissue thaws, the fluid returns to the cells, resulting in changes in volume that can rupture weakened cell walls. These combined effects are considered to be the main driving force behind the observed changes, rather than physical damage caused by ice crystals [2–5].

The outcome of the cells is highly dependent on the rate at which the tissue has been frozen. Depending on the freezing rate and the permeability of the cell, water will either flow out of the cell, which will shrink, or stay inside, forming intracellular ice crystals [6, 7].

In forensic investigations, freezing and thawing can play a crucial role in cases of unknown cause of death. Perpetrators may employ freezing preservation to conceal the body or obscure the time of death. Freezing can also occur naturally when a body is exposed to the elements, and it may even be a cause of death itself. Determining whether a body has been frozen can help corroborate or challenge a suspect’s statements and ultimately determine if any laws have been violated [3, 8, 9].

In this paper, we present a case report in which an autopsy was performed on an infant, who had died of natural causes, after undergoing freezing and thawing. The objective of this study was to reveal several histological artifacts in different tissues resulting from the freeze–thaw process. The authors compare and discuss their findings with the literature.

## Case report

### Medical history

The infant was a 1-month-old male, born at full term with anthropometrical parameters within the standard range for age: weight 3970 g, 51 cm in crown-heel length, and head circumference of 36 cm. The pregnancy was unremarkable, with normal periodic ultrasound scans, and the delivery was spontaneous and uncomplicated. Maternal vaginal and rectal swabs were negative, and she received intrapartum antibiotic prophylaxis.

Approximately 1 month after birth, the infant presented persistent cough, and aerosol therapy was promptly administered. However, after 3 days, he progressively became drowsy and unresponsive. The parents quickly took him to the hospital, but he was already in cardiopulmonary arrest. Cardiopulmonary resuscitation (CPR) was immediately started: oxygen was given through a bag valve mask, and then, an injection of adrenaline was necessary. Despite all attempts, CPR was stopped after 35 min due to pulseless electrical activity (PEA) and the infant was pronounced dead. Subsequent nasal and pharyngeal swabs revealed positive results for respiratory syncytial virus A (RSV), *Haemophilus influenzae*, and *Staphylococcus aureus*.

### Autopsy findings

An autopsy was requested to better understand the cause of death. However, in that period COVID-19 was initially spreading, and the lack of medical resources made it necessary to temporarily store the infant’s body at − 10 °C in the freezer of the morgue. Then, due to the health-related challenges arising from the COVID-19 pandemic, the autopsy was delayed for 21 days. Before the autopsy was performed, the body was thawed at room temperature (20 °C) for 12 h.

External examination showed growth parameters within the normal limits for 1 month of age: weight 4301 g (50th percentile), crown-heel length 55 cm (50th percentile), and head circumference 37 cm (25th percentile) [10]. The face was regular with no dysmorphisms. Grossly, no anomalies were identified.

Internal examination evidenced diffuse congestion of the organs, but no anatomical anomalies. The lungs displayed the expected lobes, with three on the right and two on the left. The right lung weighed 61 g and the left 50 g. Segmental sequential analysis of the heart (weight 29 g) revealed atrioventricular and ventriculoarterial concordance and regular systemic and pulmonary venous returns. The coronary arteries presented a regular course. The other organ weights were as follows: liver 173 g, right kidney 20.8 g, left kidney 22.4 g, spleen 16.4 g, brain 489 g.

**Fig. 1:**
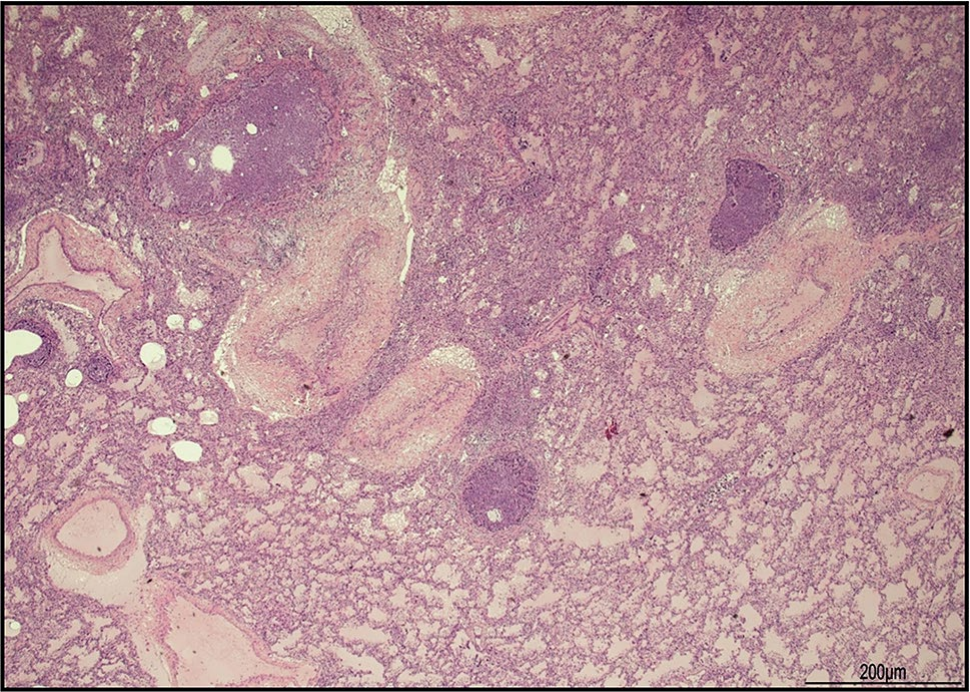
Infection of the lungs: diffuse acute necrotizing bronchitis and bronchiolitis together with pulmonary edema (hematoxylin and eosin, 2 HPF)

**Fig. 2:**
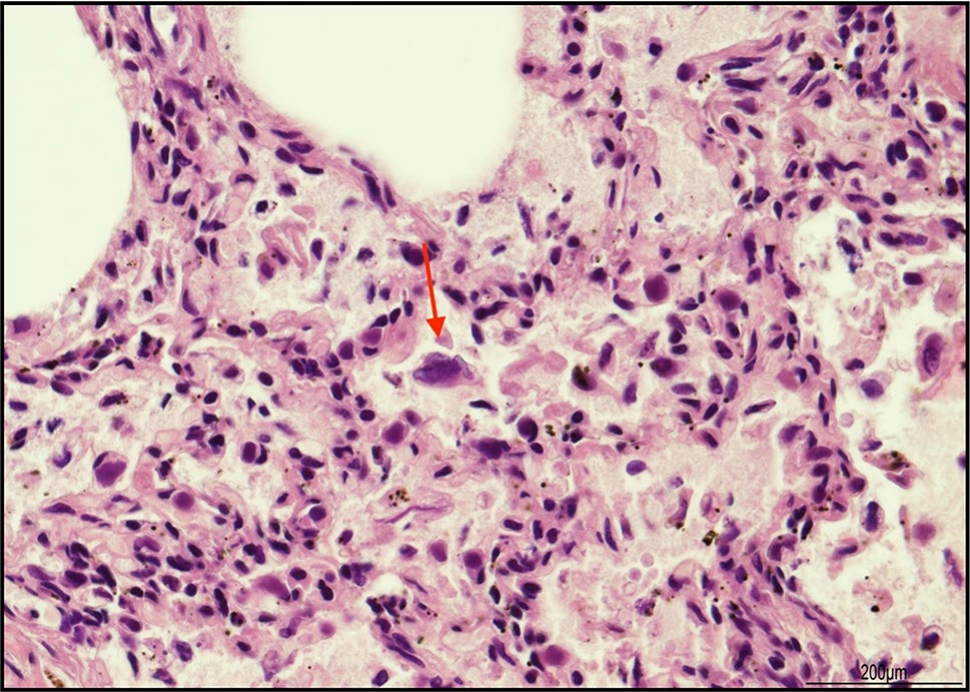
RSV infection: a sloughed off cell with enlarged and irregular nucleus compatible with viral inclusion (hematoxylin and eosin, 40 HPF)

Histological examination of the lungs revealed bilateral extensive acute necrotizing bronchitis and bronchiolitis with focal hyaline membrane formation and pulmonary edema (Fig. 1). Scattered cells with enlarged nuclei due to RSV infection were seen (Fig. 2). Minimal diffuse ischemic changes were observed in the myocardium and brain. Both kidneys showed acute tubular necrosis, tubular dilatation, and occasional microcalcifications. No significant histopathological findings were observed in the other organs.

The integration of clinical, autopsy, and histological data identified the cause of death as acute respiratory failure resulting from bilateral and diffuse acute necrotizing bronchitis and bronchiolitis. This was caused by RSV type A complicated by bacterial over-infection with *Haemophilus influenzae* and *Staphylococcus aureus*.

### Histological freeze–thaw artifacts

Evidence of histological freeze–thaw artifacts was present in many organs.

**Fig. 3:**
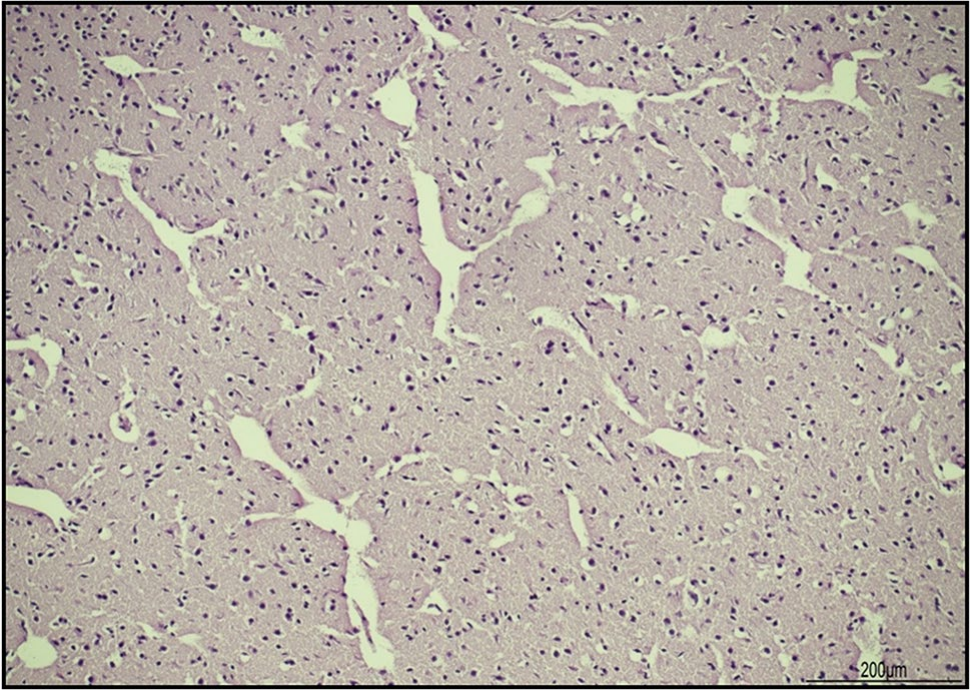
Ice crystal artifacts in the brain: the parenchyma presented multiple clefts compatible with shrinkage of the tissue (hematoxylin and eosin, 10 HPF)

The brain disclosed ice crystal artifacts with multiple clefts within the parenchyma (Fig. 3).

**Fig. 4:**
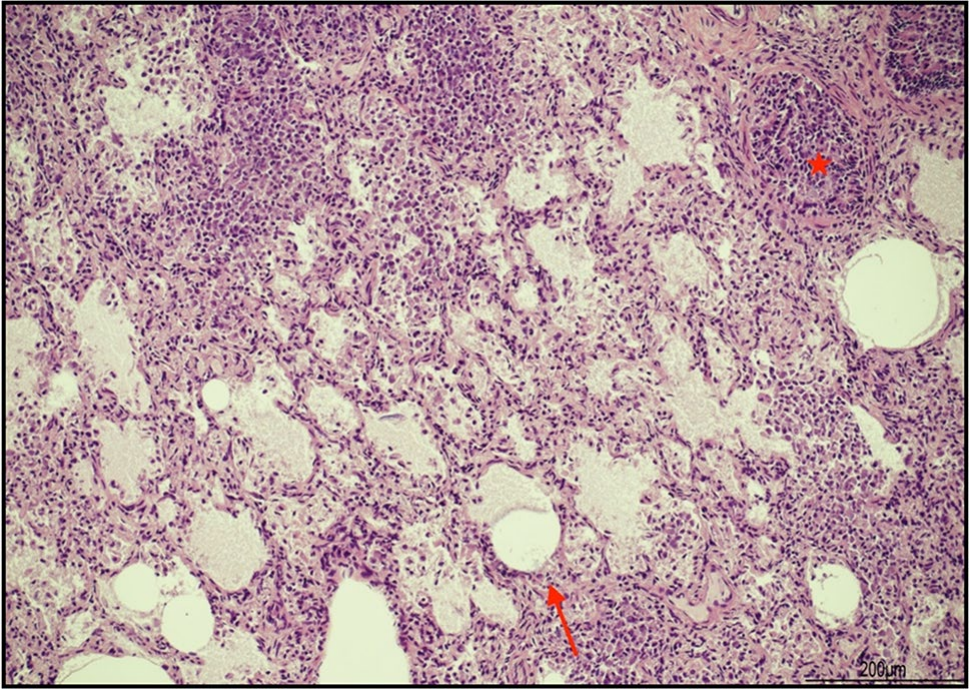
Freezing and thawing artifacts in the lungs: alveoli were overall distended (arrow); in the same field there was also evidence of bronchiolitis (star) (hematoxylin and eosin, 20 HPF)

The lungs, apart from histological evidence of infection, exhibited alveolar distension (Fig. 4).

**Fig. 5:**
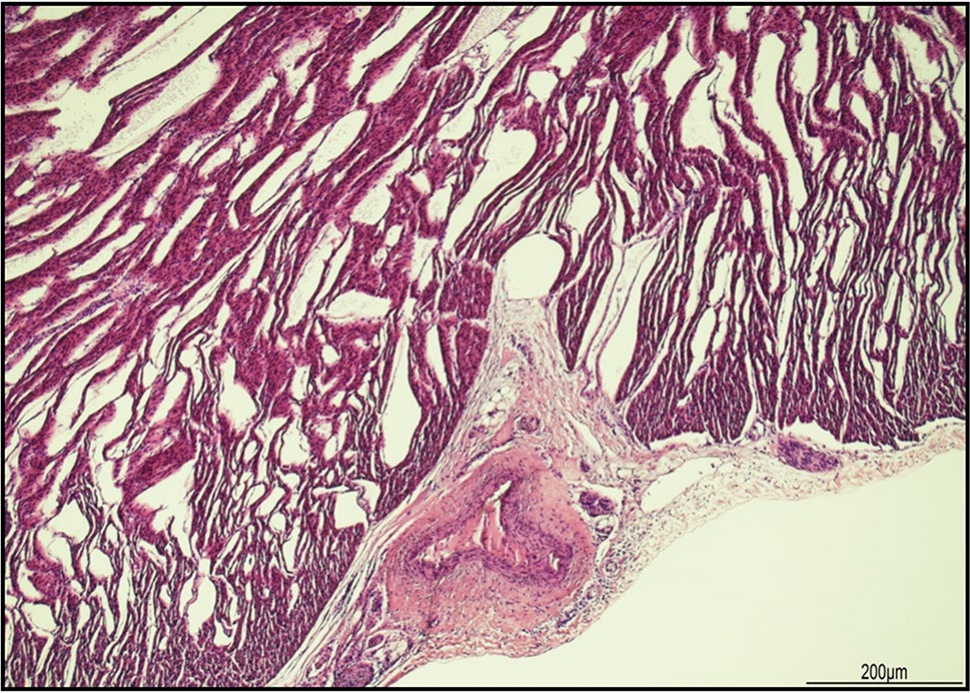
Heart: marked expansion of the extracellular space (hematoxylin and eosin, 4 HPF)

The heart presented marked extracellular separation (Fig. 5).

**Fig. 6:**
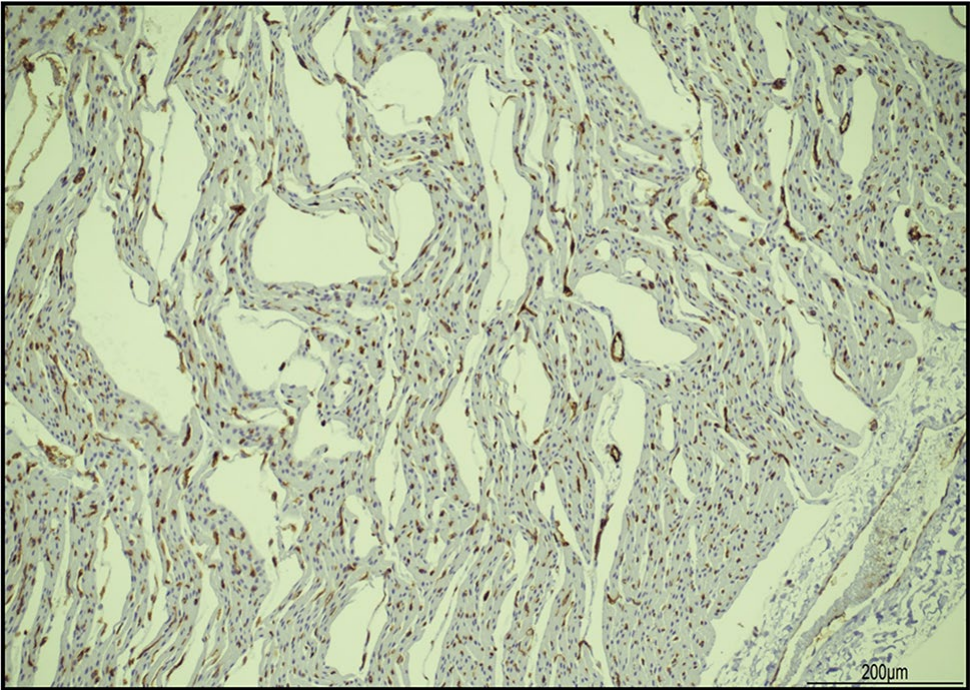
CD31 immunohistochemistry in the heart: the clefts lacked an endothelial cell lining. Small vascular structures were present between the cardiomyocytes (brown staining) (10 HPF)

Abnormal dilatation of lymphatic or capillary vessels was excluded by the regular expression of CD31, which is a marker for endothelial cells, in the vascular structures located between the bundles of cardiomyocytes. Moreover, the artifactual clefts were devoid of endothelial lining (Fig. 6).

**Fig. 7:**
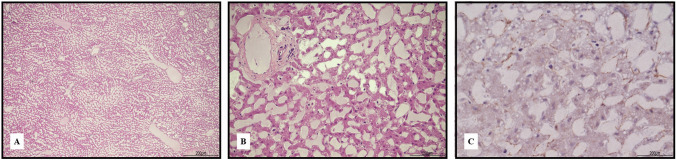
Liver freezing and thawing artifacts: **A** the parenchyma showed shrinkage of the hepatocellular laminae with marked dilatation of the sinusoidal spaces. The aspect seemed to create parallel lines similar to ice crystals (hematoxylin and eosin, 4 HPF); **B** higher magnification evidenced an unusual hepatic structure resembling hepatic peliosis (hematoxylin and eosin 20HPF); **C** but immunohistochemical expression of smooth muscle actin (SMA) was not increased (SMA immunohistochemistry, 40 HPF)

The liver showed shrinkage of the hepatocellular laminae with marked expansion of the sinusoidal space (Fig. 7).

**Fig. 8:**
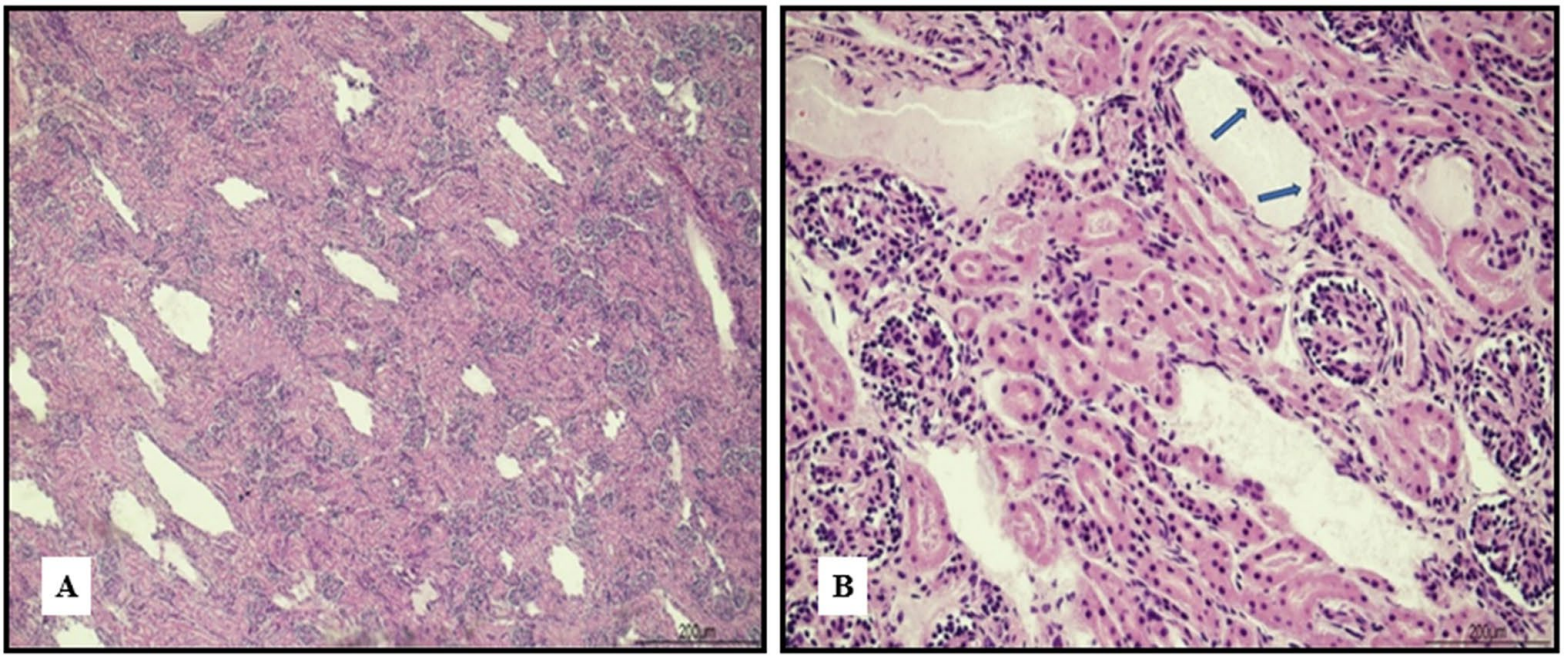
Kidney artifacts: **A** the parenchyma presented multiple cystic lacunae, resulting from the expansion of the extracellular spaces, no lacunae were lined by epithelial cells (hematoxylin and eosin, 4 HPF); **B** some of the tubules appeared stretched and compressed (hematoxylin and eosin, 20HPF)

The kidneys presented unusual cystic lacunae, probably due to expanded extracellular spaces, with tubules strained and flattened (Fig. 8).

**Fig. 9:**
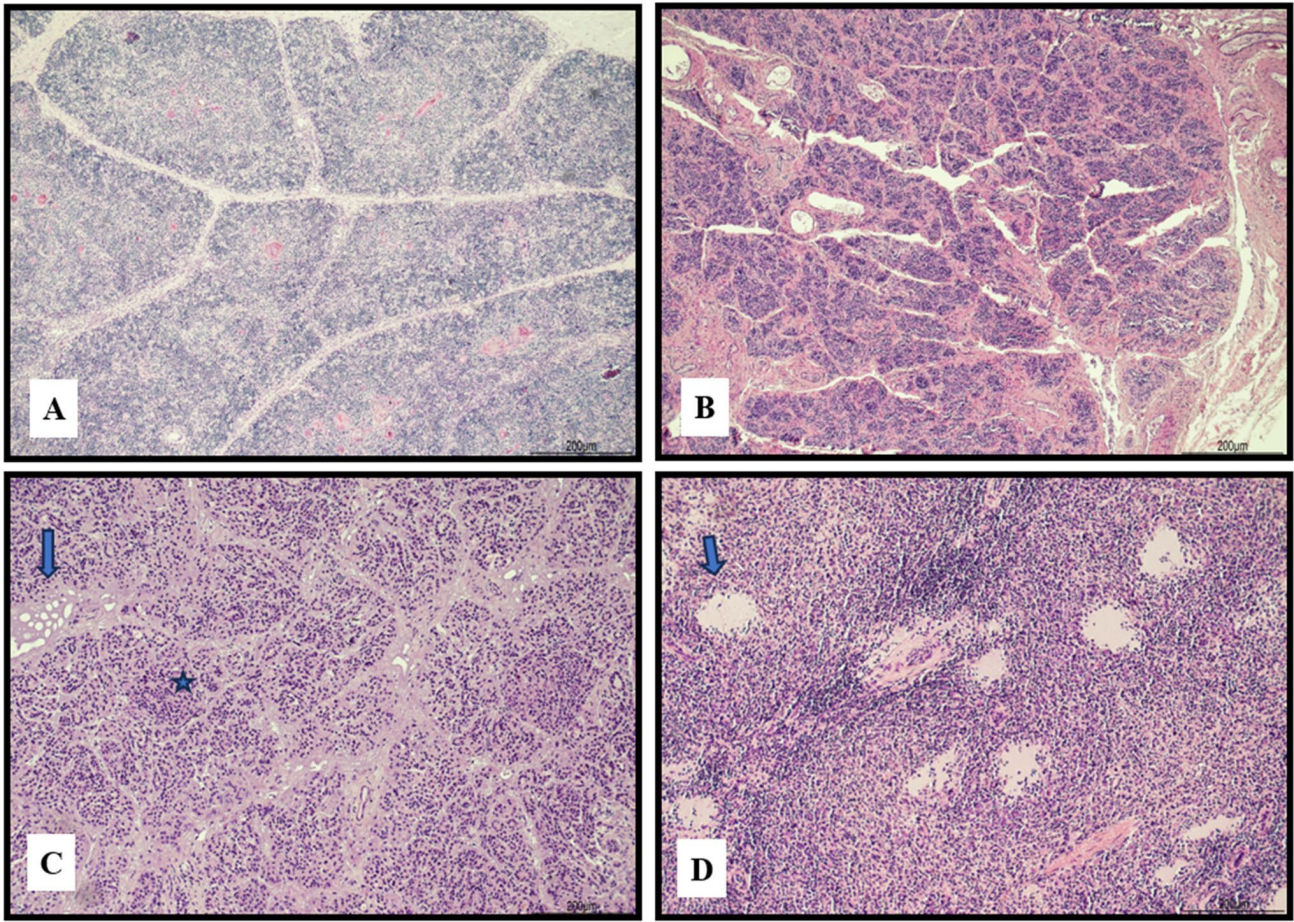
**A** Thymus: the architecture was overall well maintained with cortex and medullary division (hematoxylin and eosin, 4 HPF). **B** Thyroid: the only modifications were artifactual clefts along the septa and basophilic cellular changes (hematoxylin and eosin, 4 HPF). **C** Pancreas: the acinar structure was preserved with few lacunar alterations within the fibrous septa (arrow). The Langerhans’s islets were easily identifiable (star). All the cells were overall more basophilic (hematoxylin and eosin, 4 HPF). **D** Spleen: a few slightly eosinophilic cystic spaces devoid of an epithelial lining were the only changes observed (hematoxylin and eosin, 4 HPF)

The thymus structure was almost fully preserved (Fig. 9A). The thyroid presented clefts mostly along the septa, and the cell nuclei appeared more basophilic (Fig. 9B). The acinar architecture of the pancreas was maintained, with a few artifactual lacunae within the septa and the islets were still recognizable (Fig. 9C). On the whole, the cell nuclei appeared shrunk and strongly basophilic. The spleen displayed some cystic slightly eosinophilic spaces devoid of epithelium (Fig. 9D).

**Fig. 10:**
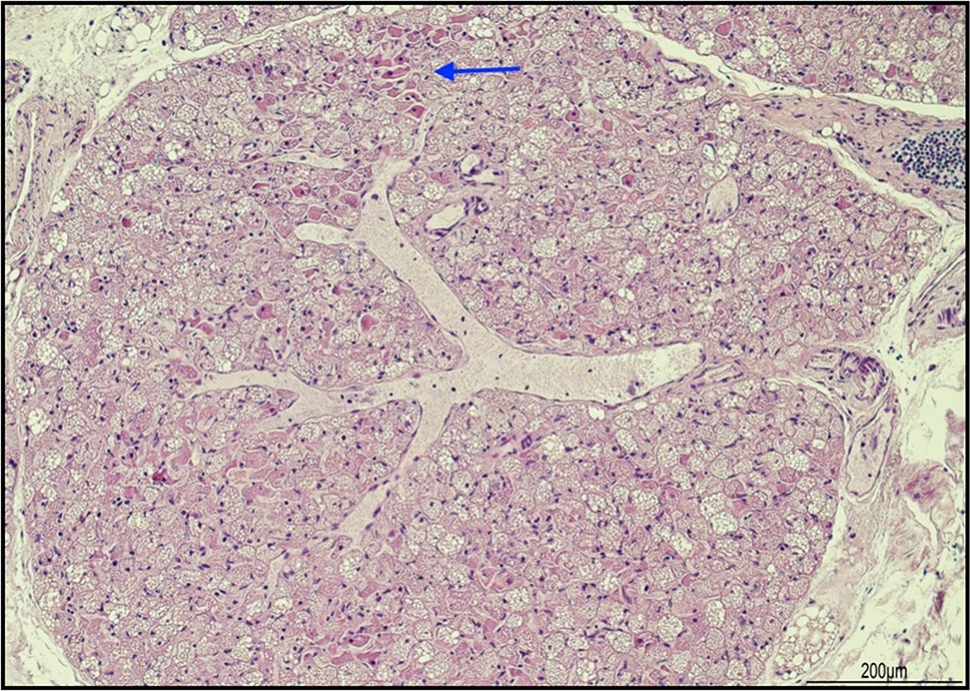
Brown adipose tissue: focal cellular shrinkage was observed in the most eosinophilic cells, the richest in cytoplasmic mitochondria (hematoxylin and eosin, 10 HPF)

**Fig. 11:**
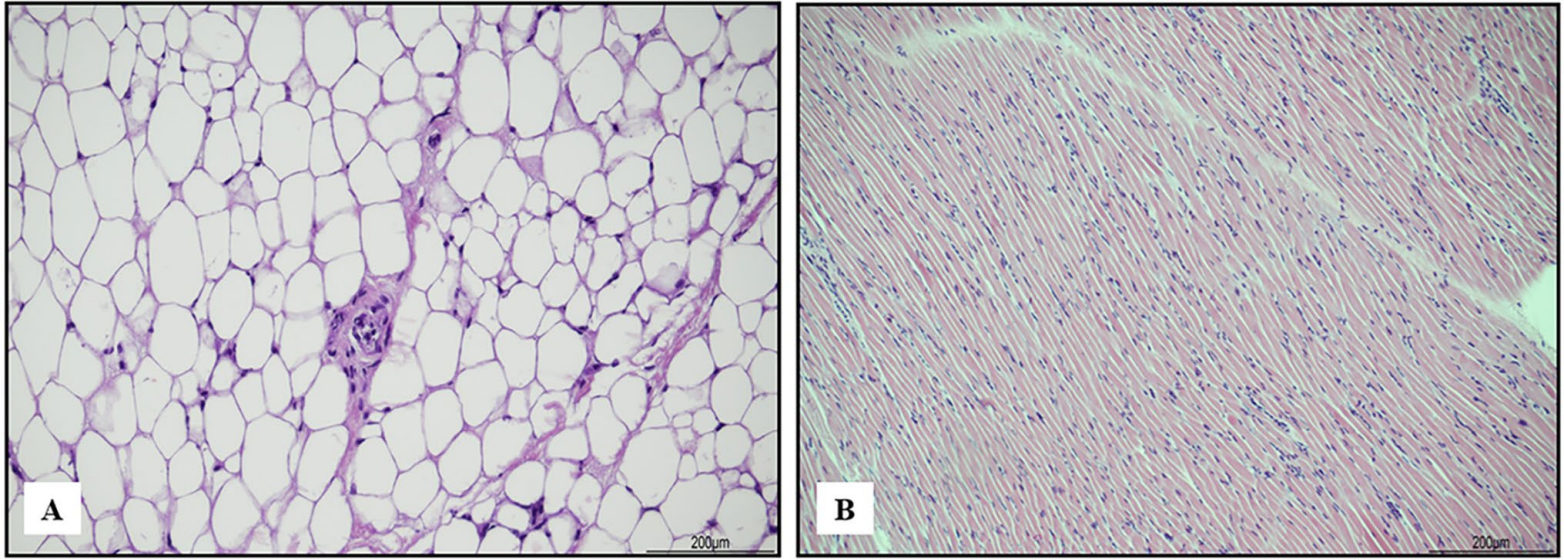
**A** White adipose tissue (hematoxylin and eosin, 20 HPF) and **B** skeletal muscle (hematoxylin and eosin, 10 HPF): the overall features were unchanged

The brown adipose tissue was mainly preserved, but focal cellular shrinkage was seen in the most eosinophilic cells (Fig. 10). The white adipose tissue and the skeletal muscle were virtually unaffected by artifacts (Fig. 11).

## Literature review

An electronic search was performed in three databases (PubMed, Scopus, and Web of Science), and keywords related to the study aim and included in the search string were (freezing OR frosty OR thawing) AND (forensic OR autopsy OR histological OR histology. The English language and time interval of publication, from January 1960 to March 2023, were applied as filters and inclusion criteria. The literature review showed that nine articles reported histological features connected to tissue freezing and thawing [3, 8, 11–17]. All the related data are summarized in Table 1 and discussed in the “Discussion” section.

**Table 1:** Cases involving an evaluation of histological features resulting from tissue freezing

Authors	Article type	Year	Subjects	Organs analyzed	Findings
Baraibar et al.	Experimental study	1985	Dogs	Brain, lung, liver, small intestine, kidney	Loss of staining, extracellular fluid accumulation, cell shrinkage, fractures, hemolysis, and hematin formation
Baraibar et al.	Experimental study	1986	Dogs	Brain, lung, liver, small intestine, kidney	Transudate, cell shrinkage, fractures, hemolysis, and hematin formation
Kagan et al.	Experimental study	2022	Black rockfish	Brain, eye, and skeletal muscle	Skeletal myocyte cavitation, lens liquefaction, and brain tissue fractures
Kozawa et al.	Case series	2010	Two newborn infants	Liver and heart	Extended extracellular areas in the heart and liver
Olsen et al.	Case report	2018	Two infants	Liver, pancreas, kidney	Development of spaces in the tissues
Schäfer et al.	Experimental study	1999	Samples of liver and heart tissue	Liver and heart	Extended extracellular spaces and shrunken cells
Pechal et al.	Case series	2017	Two infants	Brain and heart	Outline of ice crystals in tissues
Sen et al.	Experimental study	2004	Buffalo muscle samples	Muscle	The muscle fibers exhibited slight shrinkage with intermittent gaps between them
Tabata et al.	Case report	2000	Newborn infants	Skin	Karyopyknosis and vacuolation of the keratinocytes

Histological artifacts in the freezing–thawing process of tissue have not been widely reported in the literature. However, some major histological changes caused by freezing have been noted, including loss of staining, extracellular fluid accumulation, cell shrinkage, fractures, hemolysis, and hematin formation, and minor changes include loss of bronchial cilia, prominence of collagen in alveolar septa and meninges, and intracellular vacuolization of epithelial cells. In the respective studies, despite these artifacts, adequate visualization of the tissues was possible, allowing diagnosis [11, 12].

The most common histological findings in the freeze–thaw process were the expansion of the extracellular spaces and cell shrinkage, and the heart and the liver were the most widely affected organs. In general, in most tissues, strong basophilic nuclear staining and hemolysis were common [3, 13, 15, 17].

In a few reported cases of frozen newborns, cardiac and hepatic artifactual features were the most evident, with marked separation of the myocardiocytes and the sinusoidal spaces, respectively [13, 17]. In the brain, pseudobubbles or linear freeze artifacts were described [15]. In another study, skeletal muscle was reported to be preserved, but in the case of two freeze–thaw cycles, fibers underwent separation due to large crystal formation [16]. A further study showed that if a body is frozen soon after death, autolysis and putrefaction are less evident, as the cold reduces the activity of enteric microorganisms, and anaerobic decomposition is much less strong than in fresh bodies [13, 14].

## Discussion

In forensic investigations, the freezing and thawing of bodies can be critical in cases of unknown cause of death, as histological artifacts are produced in the process.

The mechanisms underlying the extended extracellular areas are still unclear [1]. Freezing of organic tissues begins in the extracellular spaces with ice crystal formation and fixing of water. Thawing melts the ice, resulting in tissue damage due to the changes in osmotic pressure between the cells and the extracellular space [3, 18, 19].

We described a case of an infant who died of respiratory failure caused by bronchitis and bronchiolitis in RSV infection. The autopsy was delayed, and the body was kept frozen in the morgue at − 10 °C for 3 weeks. It was then thawed for 12 h at room temperature (20 °C) before dissection.

The infant presented the most common histological features mentioned in the literature [3, 8, 11–17]. However, freezing artifacts in brain tissue were only mentioned in one book in our review and were described as parenchymal clefts, as we found in our case [20]. Extracellular space expansion, tissue clefts, cell shrinkage, and deep staining nuclei were found in almost every organ. Ice crystal artifacts were evident in the brain as parenchymal clefts and in the heart and liver as marked expansion of the extracellular spaces.

Freeze–thaw artifacts may also show some features of congenital diseases. The abnormal cardiac structure raised the suspicion of dilatated lymphatic or vascular channels, but immunostaining for CD31 highlighted their regular location among the bundles of cardiomyocytes. The suspicion of ventricular non-compaction was ruled out, as endocardial fibroelastosis was absent [21]. The hepatic sinusoidal dilatation could mimic hepatic peliosis, a feature of X-linked myotubular myopathy, but liver immunohistochemistry for smooth muscle actin was weakly expressed and not increased [22]. The renal lacunar cystic changes could resemble polycystic kidney disease, but these spaces were devoid of epithelial lining [23].

In our study, the adipose tissue and the skeletal muscle were the only two tissues without artifacts. Only one experimental study described artifacts in skeletal muscle, but they appeared after two freeze–thaw cycles [16]. The absence of artifacts in our case aligns with the fact that the body underwent a single freeze–thaw cycle. The effects of freezing and thawing on adipose tissue have not yet been documented. Our case exhibited white and brown adipose tissues virtually unaffected by freezing and thawing, because they are rich in fat and scarce in water, and ice crystal formation is limited.

The brown adipose tissue showed minimal cell shrinkage and deep staining nuclei, due to its composition, as the cytoplasm is packed with multiple lipid droplets and water concentration is higher than in white adipose cells [24]. Therefore, the lack of crystals should not be considered a confounding factor in interpreting freeze–thaw artifacts.

In forensic investigations, indirect indicators of freezing and thawing can provide valuable circumstantial evidence to aid forensic pathologists in understanding the sequence of events. Freezing of a body can be connected to criminal activities, such as attempts to conceal a body or manipulate the time of death, as well as natural weather conditions when the body is exposed to the elements. Ice crystal identification can offer insights into the rate at which the body froze and, when combined with other circumstantial data, becomes a significant source of information.

## Conclusions

The findings of our study can have significant implications in the field of forensic pathology. Specifically, when it is known that a body has been frozen, awareness of these potential artifacts can help forensic pathologists avoid misdiagnoses by considering the possibility of their formation. On the other hand, the presence of these alterations indirectly suggests that the body had been previously frozen and might have already thawed at the time of discovery.

## Key points

1. Freezing and thawing may disrupt the tissue architecture due to ice crystal formation and subsequent melting.
2. Histological artifacts are mostly evident in the brain, heart, liver, and kidneys.
3. Recognition of histological artifacts may help in forensic analysis whether the body is known to have been frozen or not.
4. In infants, histological freeze–thaw artifacts may mimic congenital diseases.
5. White adipose tissue is unaffected by these artifacts as it contains little water.

## Data Availability

The data presented in this study are available on request from the corresponding author.
